# “I never wanted to burn any bridges”: discerning between pushing too hard and not enough in trainees’ acts of professional resistance

**DOI:** 10.1007/s10459-024-10312-8

**Published:** 2024-02-13

**Authors:** Tasha R. Wyatt, Vinayak Jain, TingLan Ma

**Affiliations:** 1 Uniformed Services University of the Health Sciences, Bethesda, USA; 2 MedStar Health- Georgetown, Washington D.C., USA

**Keywords:** Professional resistance, Proportionality, Social harm, Trainees

## Abstract

As trainees resist social harm and injustice in medicine, they must navigate the tension between pushing too hard and risking their reputation, or not enough and risking no change at all. We explore the discernment process by examining what trainees attend to moments before and while they are resisting to understand how they manage this tension. We interviewed 18 medical trainees who shared stories of resisting social harm and injustice in their training environments. Interviews were analyzed using open and focused coding using Vinthagen and Johansson’s work, which conceptualizes resistance as a dynamic process that includes an individual’s *subjectivity* within a larger system, the *context* in which they find themselves, and the *interactions* they have with others. We framed these acts as an individuals’ attempt to undermine power, while also being entangled with that power and needing it for their efforts. When deciding on how and whether to resist, trainees underwent a cost-benefit analysis weighing the potential risk against their chances at change. They considered how their acts may influence their relationship with others, whether resisting would damage personal and programmatic reputations, and the embodied and social cues of other stakeholders involved. Trainees undergo a dynamic assessment process in which they analyze large amounts of information to keep themselves safe from potential retaliation. It is by attending to these various factors in their environment that trainees are able to keep their acts *professional*, and continue to do this challenging work in medical education.

## Introduction

When physicians and medical trainees encounter social harm and injustice in educational or clinical settings, they must make a choice; they can either ignore these harms and continue on as if nothing has happened, or choose to engage in acts of resistance to create change. While most research on resistance tends to focus on the act itself (Courpasson, 2016; Courpasson et al., 2012; Paulsen, 2015; Prasad & Prasad, 2000), in this paper, we argue that research must also consider the moments leading up to trainees’ decision as well as their ongoing reflective process during resistance. These moments, when trainees weigh various bits of information and decide whether it is worth putting themselves at professional risk, sheds light on what and how trainees ensure these acts are indeed *professional*.

Currently, not much is known about these moments, however there is some indication that resistors undergo a discernment process in which they engage in deep levels of analysis that include an assessment of various factors (Ewick & Silbey, 2003; Medina, 2013). Some of these factors may be known, such as who might be a potential stakeholder (Baum et al., 2007) what interests they may have, and what kinds of power they hold (Freeman, 1984; Vinthagen & Johansson, 2013). However, others might not be known, such as stakeholders who have yet to be identified, unforeseen roadblocks, and unanticipated consequences. In the midst of so many unknowns, understanding what goes into their decision-making and reflection process is critical because trainees need to consider how to initiate change and stay safe in a profession where retaliation is commonplace (Leisy & Ahmad, 2016). They must also navigate the tension between pushing too hard and risking their reputation, or not pushing hard enough and risking no change at all. Ellaway and Wyatt (2021) call this aspect of resistance *proportionality*, meaning trainees need to resist in proportion to their position within the hierarchy to reduce the professional risk associated with resistance.

In this study, we explore the dynamic assessment process that occurs moments before trainees decide to resist as well as the reflection process they engage in while resisting. We focus our analysis on what trainees’ report attending to because we want to support medical trainees in re-envisioning medical education through these acts. By explicating what some trainees consider in this process, we hope to provide others with critical information on how to minimize their professional risk.

## Researching acts of resistance

The literature on resistance boasts a plurality of definitions with a general agreement around two central concepts; a) resistance is an act and is always oppositional to power (Hollander & Einwohner, 2004). As such, resistance is defined as, “*a subaltern practice that might challenge, negotiate or undermine power.*”(Baaz et al., 2018)^*(*p.26)^ In each case, resistors engage in acts of rebellion that attempt to reject, challenge, or subvert power away from something/someone that has interest in maintaining the status quo for their own benefit. However, even as individuals work to critically undermine power, they are also entangled with it and need power to support their efforts in destabilizing the structures, norms, and discourses that created their subordinated position (Baaz et al., 2018).

In every act of resistance, there are two key processes resistors must engage in if they intend to be successful. First, individuals must identify what kind of power is being used against them (or others) before choosing the right form of resistance. This is because different forms of power must be met with different forms of resistance (Baaz et al., 2018). A resistor can choose from a variety of forms, but if the act is not aligned with the power being deployed, it has little chance to affect change. For example, hierarchies are a common structure that subordinates some individuals while elevating the position of others. Unlike other forms of power, hierarchies heavily depend on one group’s willingness to be obedient (Sharp, 1973; Vinthagen, 2006). However, hierarchies are not all the same. Some may be created and upheld by structures including brute force, violence, and fear of punishment, such as when the military is involved or a group is at war (Engler & Engler, 2016). In such situations, it would not make sense to resist through mere sarcasm or foot-dragging because power is being exerted functions at the level of the state and these forms of resistance will do little to create structural change.

In comparison, other types of hierarchies are upheld in more subtle ways, such as through norms and expectations, such as when individuals voluntarily place themselves in subordinated positions and surveille themselves (e.g. medical professionalization)(Baaz et al., 2018). In these kinds of situations, a revolt or riot would be inappropriate because it is misaligned with the way power is being used to maintain control. Rather,‘everyday’ forms of resistance such as noncompliance and nonconformity (Scott, 1989) may be more appropriate because they work by disrupting hidden curriculum of standardization running through the profession’s discourse (Frost & Regehr, 2013). From there, resistors can decide if they want their efforts to be large or small, public or private, overt or covert. Overt forms, include protests, riots, strikes, and sabotage, while covert forms are more hidden, such as changes in everyday repetitions, slow-work, and noncompliance (Scott, 1989).

The other key process is knowing how hard to push and the boundaries around how and what is acceptable to challenge. Every context in which resistance is enacted is different and requires individuals to assess how hard they can push against power to create change (Vinthagen & Johansson, 2013). Within the context of health professions education, Ellaway and Wyatt (2021) defined professional acts of resistance as: “*Individual and collective expressions of condemnation of social harms and injustices, with the intent of stopping them, preventing them from recurring, and/or holding those responsible to account*.” ^(p.1524)^ In the U.S., there are longstanding issues related to slavery (DiAngelo, 2016), forceful removal and intergenerational trauma among Native Americans (Brockie et al., 2021), issues of LGBTQIA + rights (Duberman, 1993), and a growing divide between the wealthy and poor (Horowitz et al., 2020), all of which are issues that can be visible in medical education. As such, trainees are known to resist issues such as racism (Zaidi et al., 2021), sexism (Blalock & Leal, 2022; Konopasky et al., 2023), and classism (Beagan et al., 2022), as well as inequitable treatment for patients and/or mistreatment of learners in the training environment. Other cultural contexts may have similar issues, but with different nuances and texture that need to be considered in the resistance process.

These two key processes, knowing what kind of power is being used to maintain control and knowing how hard to push, is essential to any resistance effort. However, these two processes have yet to be explored within the context of medical education. To study them, Vinthagen and Johansson (2013) suggest researchers explore resistor’s understanding of their *subjectivity* within a larger system, the *context* in which they find themselves, and the *interactions* they have with others. They focus on these factors (i.e. *subjectivity*, *context*, and *interactions*) because this is how power is expressed and reinforces subordination in any given situation. For example, an individual’s *subjectivity* reveals where individuals are in the hierarchy and therefore the extent to which they can create change; *contexts* reveal the boundaries constraining acts of resistance and what should be considered to minimize risk; and *interactions* reveal hidden transcripts in how individuals negotiate whom they can resist, where and how (Scott, 1990). In this study, we explore how medical trainees strategically rework power through acts of resistance in an effort to address the social harm and injustice they encounter in medical education.

## Methods

As critical scholars, our starting point for this study is that society is imbued with social structures and cultural assumptions that oppress some groups while liberating others (Kincheloe & McLaren, 2011; Steinberg & Kincheloe, 2010). Further, it is the responsibility of researchers to engage in emancipatory research towards a more peaceful and democratic society (Baaz et al., 2018). Therefore, in line with the goals of critical theory (Horkheimer, 1972), we do not seek to describe social reality, but rather reveal, critique and challenge power structures to destabilize the status quo. In this study, we attempt to do this by revealing what trainees attend to as they engage in acts of resistance in hopes of teaching other trainees how to resist in ways that minimize professional risk. The idea of *attending to* or *noticing* has been written about extensively in teacher education because what teachers *notice* in their classrooms is thought to have important consequences for their practice (Schoenfeld, 2011). What a person sees/does not see shapes what they do/do not do in the moment. In this study, we took the concept of *noticing* or *attending to* and used it to frame how trainees’ made decisions in their acts of resistance.

We followed the advice given by researchers in sociology and political science on how to study resistance, which included open-ended, semi-structured interviews to gather resistors’ experiences (Baaz et al., 2018). We framed this study using constructivist grounded theory approaches (Kennedy & Lingard, 2006) in which we iteratively revised the interview questions as we analyzed the data, tracked our understanding alongside new interviews, sought out negative cases, and continued to recruit until we reached theoretical sufficiency (LaDonna et al., 2021). Using this form of data collection allowed for the necessary flexibility to follow up on questions, redirect participants’ focus, and generally develop a broader understanding of the processes involved in the trainees’ work. In total, we recruited nine medical students and eight residents and one fellow enrolled in training environments within the U.S. and Canada during the timespan of July 2022- February 2023.

The first two participants were recruited using the first author’s personal and professional networks because they had made their resistance efforts public through explicit conversations on social media or in private conversations. Using these participants as a starting point, we switched to snowball methods for further recruiting. When potential participants ran out, we reached back into our networks and continued using snowball methods. At 12 participants, we had enough data to answer our research questions, however because every act of resistance is unique (Vinthagen & Johansson, 2013) we continued interviewing until we interviewed 18 participants to seek out discrepancies and nuances.

Each recorded video interview lasted 45-60-minutes. Interviews asked trainees about what they resisted, their experiences as resistors, who supported them in the process, as well as myriad of other questions. For this analysis, we were mainly interested in the questions of, “*When you are engaged in acts of resistance, how do you decide how hard you push? What factors do you consider in this decision-making process?”* While these questions honed in on our research questions, trainees revealed what they attended to during the process throughout the interview. Using a constructivist approach to data collection (Kennedy & Lingard, 2006), we then transcribed the audio data and analyzed iteratively to inform subsequent interviews. The video data was not analyzed. Additionally, the data analyzed for this study is part of a larger study on trainee acts of resistance (Wyatt et al., 2023). Although we used the same data set for the current study, the research questions, analytical focus, and conceptual framework shifted.

Data analysis began with open coding to understand what kinds of things trainees considered prior to and during resistance, and how they made sense of these things in relation to their goals. Once we completed open coding, we conducted focus coding by organizing the codes according to Vinthagen and Johansson’s (2013) suggestion that researchers should examine resistors’ *subjectivities*, the *contexts* in which the resistance occurred, and the *interactions* they had with others. For example, in examining *context*, we attended to both micro and macro contexts, including the immediate context in which the act of resistance was occurring (e.g., What was the issue/problem they were attempting to address, and why this was a problem?). We also attended to aspects of the broader context trainees had to weigh pros and cons against (e.g., What long-term contexts did they consider in relation to their immediate acts?). In *subjectivities*, we took on the perspective of the trainees and focused on concerns the trainees had about themselves and others (e.g., What is my position in this system? What might be the consequences of my actions?). In *interactions*, we focused on what trainees noticed about others before deciding to resist (e.g., What did they observe about those they were interacting with? How did they interpret these interactions and extrapolate the meanings to help situate their acts?). In each case, our goal was to take on the perspective of the trainees to understand what they were attending to moments before making the decision to resist and during the process of resistance. We then analyzed the results in relation to the kinds of power trainees were resisting, which included such things as racism, homophobia, mistreatment of patients and learners, as well as the organization of the medical profession itself.

Each team member identifies as a critical scholar working in medical education, and therefore brings their backgrounds, ideologies, and allegiances to the study of trainees resistance (Thompkins & Leonhard, 2017). The team consisted of a white critical researcher (TW) who is interested in the boundaries around professional resistance. She is interested in which lines can be crossed in medical education, even if the social norms tell you they cannot, and which ones are considered unforgivable. The second author is a resident physician (VJ) studying critical pedagogies who resists social harm and injustice in clinical settings. In his leadership role, he encourages his residents to engage in resistance, but wants them to do so safely. He is interested in where and how safety is jeopardized. The third author is a social science researcher (TM) who studies aggression, mistreatment, and burnout. She is interested in the complex system that allows resistance to emerge, including the professional identities of resistors themselves. This study was deemed exempt by Uniformed Services University under IRB #DBS.2022.344, approved May 21, 2022.

## Results

When deciding on how and whether to resist, trainees underwent a cost-benefit analysis weighing the potential risk against what chance they might have to create change. Trainees were particularly concerned about how their acts may influence their relationship with others (e.g., supervisors, ability to achieve their goals within other departments, etc.). They also considered their program’s tolerance level, especially around student activism, and whether resisting would damage their own and their program’s reputation. Additionally, trainees maintained an ongoing assessment of the subtle messages that were communicated within their interactions with others, including the stakeholders’ (who sometimes can be opponents) body language, the level of strain in their voice, and other social cues. In short, trainees took in large amounts of information, assessed them in relation to one another, and then decided the best course of action to address the social harm and injustice they encountered. Below, we organized the results by what trainees attended to in the *context* where they were resisting, trainees’ *subjectivity* within the larger system of power, and the *interactions* with others they considered in the decision-making process.

### Assessing context: *“There is kind of a cost-benefit system going on... in my head”*

In determining how hard to push, trainees indicated that this decision was very personal and individualized, and that there was no set of rules to follow. Rather, they engage in an ongoing analysis of various factors that requires them to consider large amounts of information. Several described this process as a “cost-benefit analysis” in which they identified what issues they were interested in, and how likely they were to see change if they resisted, as this resident described, “[Trainees have] to know what things to push for and what things not to push for” (Resident -P9). Part of this process included being realistic about whether they could achieve their desired outcomes, and if change was not possible, knowing when to back off. As one resident explained, “In those instances where it’s obvious you will lose... it’s better to just not engage” (Resident-P6). For example, if the Dean did not find it important for students to be well trained in LGBTQ care, students might not directly target their leader, but rather turn to “teaching our classmates, who are going to be the new providers of the [next] generation, [which] is going to be a lot more impactful” (Resident -P6). However, if there was even a small chance that their act of resistance might create change, participants will resist directly and in doing so described the importance of identifying the right “time and place... to bring things up” (Student-P17). For example, the following student described their process of weighing the benefits against the risks before deciding to resist,

There’s kind of like a cost-benefit system going on, where in my head I had to calculate what I would say and where I would say it. I could be in the right for a lot of things, [but] if I brought it up in the wrong settings, then I could be perceived as a complainer... [and] I could lose my credibility. (Student -P17)

Although many trainees talked about waiting for the right “time and place”, most of the racially minoritized trainees expressed that it was not feasible to do so because their well-being was always in jeopardy. This resident explained that he did not have this luxury because as a Black physician, there is always too much at stake for him, his Black colleagues, and his Black patients. He does not attend to timing at all, rather he examines how egregious the infraction is as part of his assessment, “It’s based on... the urgency of the issue, how close you are to the issue, in terms of it personally affecting you, and how much that effect really inhibits your ability to succeed” (Resident-P16). In some cases, when trainees could not discern whether the time was right, they looked to any hints of “past precedent” to make a decision, as this resident explained, “I would see what... in the past had caused people to get into trouble and caused people to get kicked out” (Resident-P5). Relying on what had worked and not worked in the past was one strategy trainees used as they attempted to discern whether resisting was worth the risk.

Each participant explained that finding this line was incredibly important, because they were concerned about their personal safety and “potential retaliation or possible consequences” (Resident-P9). In particular, trainees feared their administration most of all, because they believed that by pushing too hard, they could get expelled of medical education, and then be forced to pay back their loans without a means to do so. As one resident explained, it seemed like administrators were just looking for excuses to get rid of problem students, “They will find any reason to not graduate you... [which] is the worst thing that can happen to somebody who wants to be a doctor” (Resident-P13). The fear of retaliation was ever present in the decision-making process, and each trainee told stories about the potential consequences waiting for them if they pushed too hard. For example, some described peers they knew or had heard about who had been retaliated against, such as this resident, “I have heard of instances where that has happened and so that’s why I’ve also been cautious” (Resident-P6). Or, trainees had been retaliated against themselves and were afraid of experiencing it again, “A sense of personal safety is like a newer thing that I’ve developed that keeps me from *overtly* [resisting]” (Student-P18). This fear of retaliation encouraged them to oscillate between thinking about their own safety in the moment and their safety at a future point.

In fact, knowing that those more powerful than they could retaliate kept trainees watchful for this invisible line between pushing too hard and not enough. As such, they constantly attended to changing circumstances that would provide clues as to whether it was safe to resist. In one case, a resident explained that she would not sign a petition that her peers were organizing because she was terrified that it might come back to harm her, “I knew if my name was attached, I would be black booked from my school [which has] a history of [the administration] treating you poorly, not giving you letters of recommendations, doing anything they can to prevent your further education” (Resident-P6). Others described knowing that they had crossed the line, but realizing it too late, such as this student who recalled a time when she resisted and it went poorly, “I [can] feel myself kind of going back to that moment, those moments on rounds when I was like, ‘Ooh, everyone disagrees with me and I’m still talking’” (Resident-P14). Therefore, in the cost-benefit analysis that participants described, assessing the context for professional safety in the moment and in the future was cited as the “first and foremost” (Student-P18) consideration in the decision-making process.

### Assessing Subjectivity: “*I’ve never wanted to burn any bridges”*

In deciding whether to resist, trainees considered their position in the larger system, what consequences they may experience, and the relative gain they could make. For example, trainees discussed whether an act of resistance may affect future opportunities that might be available to them. Thinking about the future was a critical aspect because trainees didn’t want to limit themselves as they attempted to create change, “I never wanted to burn any bridges” (Student-P17). They also assessed how important the issue was to them and if it would affect their ability to continue resisting at some future point, “If I push now, can I push it later?” (Resident-P12). In each case, they attempted to find the line between pushing too hard and not enough, which was succinctly described by this resident, “I would set very harsh boundaries for myself so I can’t go beyond this [line] in my act of resistance because now it’s going to kick me out” (Resident-P5).

In situations where trainees held a title, such as chief resident, they found that even though “there are limits to resistance,” their official titles and their “weight” provided a modicum of privilege to initiate change that they used for the benefit of others (Resident-P8). For example, one student, who was a class president, indicated that they were not afraid of confronting those in power because he felt justified given the harm they committed, “It [was] within my own right. I stayed within my lane and within my channels” (Student-P17). As he explained, his position gave him authority to push back on those with power without feeling afraid, “Being class president afforded me a different avenue to be able to pose questions, ask uncomfortable questions to admin, and bring different discussions to the forefront. I knew where I stood” (Student-P17). However, even trainees with credentials still had to be careful, lest they risk losing this privilege and having to resist without formal protection, “Not everybody gets to resist like that. I get to do it because I have this professional title and if I lose [it], I lose the ability to protect people” (Resident-P8).

Residents and fellows, involved directly with patient care, took into consideration how their acts might affect relationships within other departments, which could have dire consequences for patients. For example, one resident routinely asked herself the following questions before resisting,

Am I representing my department well in this interaction? Will [it] change the types of things that people are willing to do for our department [in the] future? Will this interaction ostracize me or us from this person or this group? If I push now, can I push later? [I think about it] as the overall strategy for whatever I’m trying to achieve. (Resident-P12)

In other words, trainees assessed the relative gain of each act of resistance. They considered not only the effect it may have in the moment, but also considered how their position might affect others in assessing whether to resist. Further, they attempted to maintain a flawless reputation so that when they did resist, their acts of resistance would be taken seriously, “If you’re going to be resistant, you also have to be impeccable. You can’t allow other avenues of discreditation” (Resident-P9).

Yet, due to racism, racially minoritized trainees expressed that maintaining an impeccable reputation was incredibly challenging given the stereotyping they experience. For example, one trainee expressed that there are certain “aspects of your identity [that always] put you at risk” (Resident-P16) and therefore it is always risky to resist. This trainee expressed that he could not think of his reputation in these moments because often he felt there was often no other option other than to resist. When he experienced social harm and injustice, his reaction was always to “Sound the alarm, [because] I am going to have to fight. I am going to have to find a way or make one” (Resident-P16). In other words, when you are racially minoritized, the stakes associated with not resisting are just as dangerous as the risk of resisting.

### Assessing Interactions: *“[I had to do] careful word surgery”*

As part of the cost-benefit analysis, trainees considered the individuals they were confronting because it is important **“**to know who you’re working with” (Resident-P10). They described looking for “social cues” (Resident-P9) in other people, such as reading their “body language and their tone” (Resident-P12). Having ongoing experiences with administrators and key stakeholders helped trainees to assess other individuals because it helped them get better at assessing whether they can resist without retaliation, as this resident explained, “work[ing] with a lot of the same people over and over, you can kind of read them after a while” (Resident-P12). Others explained that they relied on their own experiences to assess who can be targeted. After years of experiencing social harm and injustice in society, as one racially minoritized resident explained, it becomes easier to identify who can be confronted, “When you’ve been exposed to this kind of stuff for so long, especially as a minority in medicine or just a minority in general...you kind of get better at gauging who’s who” (Student-P15). Trainees explained that the ability to read body language and social cues is critical to knowing how hard to push. Further, those who are not skilled at this assessment often suffer for their oversight, “The people that truly get kicked out of the system are the ones that were sometimes even right, but they were oblivious to who they were talking to, or they weren’t reading the social cues as well” (Resident-P10).

Building capacity for change via identifying key supporters was important in addition to resisting social harm and injustice in the educational and clinical environment. As this student explained, “Building capacity involves working with faculty because...having that support makes a big difference” (Student-P14). Trainees indicated that identifying key faculty to support trainees was part of the assessment process, although not all faculty members had potential as co-resistors. Trainees looked for faculty members who were interested in creating change, but may not know how to go about it. Participants explained that these are the individuals to focus their work on because these faculty are in positions of power to create meaningful change. Again, trainees need discernment identifying individuals who are prone to help and are “more open to a conversation” (Student-P15). In asking for a fuller explanation on how to discern an open faculty member from another, the trainee expanded on what this looks like,

People who make comments in a very dismissive way tend to be the ones I feel like, ‘All right, like you’re just kind of in this fixed mindset (chuckles) and nothing I say is going to change that, and if I say something, you might come after me next’. Then there are other ones who are curious, and it sounds less hateful, [but] just more ignorant. You can tell the difference in the way somebody says something... [and] I’m not going to waste my time on [them]. (Student-P15)

After assessing embodied and linguistic cues of those involved, trainees spend an inordinate amount of time crafting the delivery of their words because, as they said, “delivery is important” and how a message is delivered affects the outcome. For example, one student indicated, “It is important that we deliver things appropriately as best we can because then it gives us more weight when we’re trying to push harder for other aspects of change” (Student-P4). In preparing to resist, some sought out multiple points of feedback and rehearsed their message before delivering it, “Everything that I wanted to say, I made sure that I said it in the exact way I wanted it to be perceived” (Student-P17). In describing this process, one student indicated,

It’s very political. You really have to be tactful and almost manipulative to get things done, which is not ideal. It was like everything that you said had to be carefully thought out and do careful word surgery to get the... point across without offending people, which was very, very exhausting. (Student-P19)

## Discussion

Any act of resistance aiming for more inclusive educational and clinical environments accompanies a serious risk to one’s personal and professional standing. However, despite this, medical trainees are willing to place themselves in this precarious position to address the social harm and injustices that they encounter. This study demonstrates that trainees do not decide to resist thoughtlessly. Rather, they do so intentionally, attending to various factors in their environment to mitigate the potential risk of retaliation and identifying the line between pushing too hard and not enough. These factors include the elements found in their micro and macro *contexts* (e.g., What should I consider in this situation to mitigate the potential risk?), their *subjectivity* within the situation (e.g., What are the potential consequences of my actions given my current position?), and evaluating the *interactions* they have with others (e.g., How open is this person to receiving and responding to my act of resistance?). Their consideration of these factors is what makes these acts of resistance *professional*. Trainees meticulously attend to the dynamic interaction of these three factors and then act within the constraints of the system (i.e. medical training) so that they may continue to do the challenging work that resistance entails.

Although these factors (i.e., *context, subjectivity*, and *interactions*) have been used to study resistance elsewhere (Vinthagen & Johansson, 2013), they have yet to be applied in a professional setting like medical education. Studying resistance in this profession is a unique contribution because trainees undergo tremendous amounts of risk in breaking with medicine’s chains of obedience. Deference and obedience is an integral aspect for how medicine is structured, so much so that it is easy to forget that trainees’ submission to the hierarchy is a choice (Sharp, 1973). In fact, the structure of the medical profession relies on individuals making this choice repeatedly, which ultimately maintains the profession’s organization. With each act, trainees recommit themselves to the profession as they work to shape it into one that is fair, equitable and safe for everyone. In fact, safety was a particularly strong theme among the racially minoritized trainees whose experience with resistance is one of survival for their own selves and their patients. Given the spotlight on the importance of safety for marginalized individuals in society and medicine (Taiko et al., 2021), acts of resistance will become increasingly important to examine. We can no longer think of them as single, disconnected events, but instead they are part of a much larger social movement in medicine and medical education.

While many of the findings substantially contribute to the resistance literature, one key finding for medical education is that trainees described actively searching for faculty members who have the potential to work alongside them. This raises the issue of how educators can help trainees stay safe as they engage in acts of resistance in both educational and patient care environments. While future work is needed on what strategies faculty members can use to support trainees, there seem to be several opportunities for faculty to assist with this process. First and foremost, creating an open-minded conversation and listening carefully for trainees’ concerns and making them feel heard is imperative for building positive working relationships. Additionally, faculty could help to build mental preparedness around the kinds of social harm and injustice trainees might encounter in a particular setting. For example, attendings might explicitly acknowledge that there will be issues needing resistance and potentially set up an orientation at the beginning of a rotation or residency where they bring these issues out into the open and discuss them with trainees.

Faculty might also provide structured opportunities for trainees to voice their concerns before the situation escalates thus thwarting trainees’ feelings that they have no other choice but to resist. This might entail discussions at regular intervals on some of the barriers that trainees are experiencing to provide the best care possible to patients or improve the educational environment. Acknowledging the challenges trainees experience and providing opportunities for them to think through how best to resist would ensure everyone functions as a unified whole. Faculty may also want to explicitly help trainees balance resistance with the risk they may encounter. For example, rather than letting trainees figure out what to attend to in resisting, faculty could teach trainees how to effectively consider *context, subjectivity*, and *interactions* in decision-making. In all of these suggestions, the idea is that faculty members should begin to normalize resistance so that trainees can achieve their desired outcomes without the real or imagined threat of retaliation. And yet, even better solution for how to normalize resistance in medical education is for faculty to join trainees in these acts. Creating wide-spread change against social harm and injustice should not be left to trainees alone, but should be a collective effort in medical education.

While this study effectively gathered data on the moments before trainees decide to resist, and while they were in the process, this study is limited in understanding other aspects of the decision-making process. For example, individuals are thought to respond to the dynamic and changing circumstances with each bit of information that is included in the decision-making process. Meaning, if the context shifts or a new person enters the situation, individuals undergo a new and altogether different process of assessment. Thus, resistance is always situational and contextual and needs to be sensitive to larger historical, economic, and political shifts (Mumby & Robyn, 2017). We did not ask about how new information may shift their decision making in the moment. Rather, we focused on what factors were considered in their decisions.

Additionally, this study did not focus on when trainees decided *not* to resist, which is equally important. Previous research shows that individuals also need to recognize that some things matter more than others in a conflict (Courpasson, 2016). They have to wisely discern what to give up momentarily in exchange for long-term gain. This points to the fact that when resisting you have to let some things go and focus on other things that help advance the cause (Courpasson, 2016). It might be important to examine why trainees choose not to resist on certain occasions as much as it would be to assess what is considered in the decision-making process. And finally, we did not study resistance over time, which could be useful in demonstrating that resistance is a set of acts that can significantly transform organizations over longer periods (Courpasson et al., 2012). Previous research shows the strategies that resistors choose changes over time because it has to adapt to the changes of power (Vinthagen & Johansson, 2013). Future work might want to conduct longitudinal studies to examine trainees as they work to shape their educational and clinical environments.

However, despite these limitations, this study adds to our understanding of the work that trainees are doing to create change within medical education. We know that the trainees of today come from a long line of others who dissented against institutional repression and entrenched professional structures (Chowkwanyun & Howell, 2019). One important contribution is that this study leaves a permanent record of what trainees were doing to resist at this point in time for others to build upon. Further, as Vinthagen and Johansson remind us, resistance should always be thought of as a dynamic, creative, and interactional process between those who rule and those who rebel. Those in power will always find ways to control and create obedience, and those who are made to be obedient will also find new methods and strategies to fight back. This is because each generation evolves by building on past mistakes and successes. As researchers, we must continue to study these acts as they are unfolding. As a community, we need records of these acts for future generations to know how courageous these trainees were in reimagining the next iteration of medical education.

## Data Availability

The datasets generated during the current study are not publicly available due to the high risk involved in exposing participants and their institutions.

## Declarations

### Disclaimer

This work was prepared by a civilian employee of the US Government as part of the individual’s official duties and therefore is in the public domain. The opinions and assertions expressed herein are those of the author(s) and do not necessarily reflect the official policy or position of the Uniformed Services University or the Department of Defense.
